# Genome-wide identification and expression analysis of *CCoAOMT* genes in *Capsicum annuum* L. under drought stress

**DOI:** 10.3389/fpls.2025.1654390

**Published:** 2025-09-19

**Authors:** Di Wu, Chen Lu, Liwei Bai, Xi Yan, Wei Lai, Xiaoming Zhang, Lei He, Jianwen He

**Affiliations:** ^1^ Pepper Research Institute, Guizhou Academy of Agricultural Sciences, Guiyang, China; ^2^ Guizhou Key Laboratory of Molecular Breeding for Characteristic Horticultural Crops, Guiyang, China; ^3^ Guizhou Mountain Agricultural Machinery Research Institute, Guiyang, China

**Keywords:** *Capsicum annuum*, *CCoAOMT* gene family, drought stress, expression, whole-genome identification

## Abstract

**Introduction:**

Lignin biosynthesis is critical for plant structural integrity and stress response, with *Caffeoyl-CoA O-methyltransferase (CCoAOMT)* playing a pivotal role. This study investigates the *CaCCoAOMT* gene family in pepper (Capsicum annuum) based on the Zunla-1 genome to elucidate their molecular characteristics and drought stress responses.

**Methods:**

Eleven *CaCCoAOMT* genes (*CaCCoAOMT1*–*CaCCoAOMT11*) were identified and analyzed for physicochemical properties, phylogenetic relationships, gene structure, conserved motifs, and promoter elements. Gene expression patterns were validated using qRT-PCR under drought stress, and subcellular localization of *CaCCoAOMT1* and *CaCCoAOMT2* was determined in tobacco leaves.

**Results:**

The *CaCCoAOMT* genes are distributed across chromosomes 1, 2, 4, and 8, with *CaCCoAOMT10* and *CaCCoAOMT11* unanchored. The encoded proteins range from 143 to 380 amino acids with 2–10 exons. Phylogenetic analysis classified the genes into clades II, III, V, and VII. Ten conserved motifs were identified, with motifs 1 and 2 present in all genes. Promoter analysis revealed cis-elements responsive to light, hormones, and drought stress. Expression analysis showed tissue- and developmental stage-specific patterns, with all genes except *CaCCoAOMT6* exhibiting differential expression. Under drought stress, six genes were significantly downregulated and two were upregulated in roots. *CaCCoAOMT1* and *CaCCoAOMT2* localized to both the cytoplasm and nucleus.

**Discussion:**

These findings highlight the structural and functional diversity of the *CaCCoAOMT* gene family and their regulatory roles in drought stress response in pepper. The differential expression and subcellular localization suggest specific roles in lignin biosynthesis and stress adaptation, providing a foundation for further functional studies and potential applications in improving drought tolerance in pepper.

## 1 Introduction

Pepper (*Capsicum annuum* L.) is a globally significant crop, but its cultivation is hindered by drought stress, which adversely impacts yield and quality (Zheng et al., 2024; Zhou et al., 2024). A key plant response to drought is the alteration of lignin biosynthesis, crucial for enhancing drought tolerance (Moloi and Ngara, 2023). Lignin, a complex phenolic polymer in the plant cell wall, is vital for structural integrity, mechanical strength, efficient water transport, and environmental stress response mechanisms (Liu et al., 2018; Han et al., 2024). Studies across various species have highlighted the importance of lignin in drought tolerance. For example, in rice and grapevine, the activation of lignin biosynthetic genes enhances drought resistance by improving root structural integrity and maintaining photosynthetic efficiency (Bang et al., 2022; Wang et al., 2022). Similarly, in maize and cassava, lignin accumulation contributes to drought resistance by modulating stress-related pathways and oxidative stress responses (Tu et al., 2020; Bang et al., 2022). Lignin is a complex phenolic polymer deposited in plant cell walls, particularly in vascular tissues like xylem. This deposition provides structural support, allowing plants to maintain upright growth and withstand mechanical stress during drought conditions. The rigidity imparted by lignin is essential for maintaining cell wall integrity under water-deficit scenarios (Jiao et al., 2024). The hydrophobic nature of lignin decreases cell wall water permeability, thereby reducing water loss through transpiration. This property is particularly beneficial under drought stress, as it helps plants conserve water and maintain cellular hydration (Han et al., 2022). Lignin is integral to the formation of the Casparian strip in the endodermis, which regulates the uptake of water and solutes into the plant. Additionally, lignin deposition in xylem vessels enhances the plant’s ability to conduct water over long distances, ensuring adequate hydration during periods of limited water availability (Yadav and Chattopadhyay, 2023). These findings collectively highlight the importance of lignin biosynthesis in enhancing plant drought tolerance by improving cell wall structure, regulating water movement, and reducing water loss under stress conditions.

Lignin biosynthesis is a multifaceted process that involves a variety of enzymes and metabolic pathways (Choi et al., 2023; Han et al., 2024). Among these, the *CCoAOMT* genes are pivotal, as they participate in both lignin and flavonoid synthesis within the phenylpropanoid biosynthesis pathway, thereby contributing to plant resistance mechanisms (Kim et al., 2007; Gu et al., 2010). Caffeoyl-CoA O-methyltransferase (*CCoAOMT*) is a pivotal enzyme in the lignin biosynthesis pathway, catalyzing the methylation of caffeoyl-CoA to produce feruloyl-CoA, a precursor for guaiacyl (G) lignin monomers. This methylation step is crucial for the formation of lignin, a complex polymer that provides structural support and resistance to environmental stresses in plants (Yadav and Chattopadhyay, 2023). In maize, the *CCoAOMT* gene is essential for lignin biosynthesis, significantly influencing lignin composition and the structural integrity of the plant cell wall. Alterations in *CCoAOMT* genes have been shown to modify lignin content and composition, which in turn affects plant degradability and mechanical strength (AFornalé et al., 2016). In the fern *Polypodiodes amoena*, two *CCoAOMT* genes have been identified and functionally validated, demonstrating their capacity to methylate caffeoyl-CoA and contribute to lignin biosynthesis when expressed in *Arabidopsis thaliana* (Zhang et al., 2019). Beyond its role in lignin formation, *CCoAOMT* is also involved in plant defense mechanisms. In maize, *CCoAOMT* interacts with hydroxycinnamoyltransferase (HCT) and the NLR protein Rp1 to modulate immune responses (Wang and BalintKurti, 2016). In the hybrid species *Acacia auriculiformis × Acacia mangium*, the enzyme *CCoAOMT* plays a critical role in modulating lignin content and composition, which has significant implications for wood quality and industrial applications such as pulp production (Pang et al., 2014). Additionally, *CCoAOMT* is involved in regulating carbon flux between lignin and other phenylpropanoid-derived metabolites. For instance, in *Asarum sieboldii*, altering *CCoAOMT* expression shifted the metabolic balance towards increased phenylpropene production at the expense of lignin synthesis under specific conditions (Ji et al., 2023). These findings underscore the pivotal role of *CCoAOMT* in plant metabolism and its potential as a target for genetic engineering aimed at optimizing lignin content and composition for diverse applications (Guo et al., 2019; Lam et al., 2019). Consequently, *CCoAOMT* emerges as a key regulator in lignin biosynthesis and plant stress adaptation across various species.

Extensive research has been undertaken to identify and characterize the *CCoAOMT* gene family across a diverse range of plant species, underscoring its importance. In jute (*Corchorus* spp.), a comprehensive genome-wide analysis has elucidated the structural, functional, and evolutionary attributes of *CCoAOMT* genes, alongside their expression profiles under abiotic stress conditions (Kahie et al., 2023). Similarly, in the tea plant (*Camellia sinensis*), ten *CCoAOMT* genes have been identified, exhibiting conserved gene structures and motifs, which offer insights into their phylogenetic relationships and potential roles in lignin biosynthesis and stress responses (Wang et al., 2024). Furthermore, *CCoAOMT* gene families have been identified in various other plant species, including *Gossypium*, *Solanum tuberosum*, *Dendrocalamus farinosus*, *Populus*, *Malus domestica*, *Pyrus bretschneideri*, and *Prunus persica* (Li et al., 2021; Zhao et al., 2022; Wei et al., 2023; Ma et al., 2024; Peng et al., 2024). Despite recent advances, a comprehensive identification and evolutionary analysis of *CCoAOMT* genes in pepper has not yet been undertaken. In this study, we utilized bioinformatics methodologies to systematically identify and characterize the *CCoAOMT* gene family in pepper. Our analysis encompassed the examination of protein physicochemical properties, evolutionary relationships, gene structures, cis-regulatory elements, and gene duplication events. Additionally, using qRT-PCR analysis, we identified candidate *CaCCoAOMT* genes that are responsive to drought stress. This research not only enhances the genomic information available for the *CaCCoAOMT* gene family but also lays the groundwork for future investigations into their functional roles in lignin biosynthesis, stress adaptation, and potential applications in crop improvement.

## 2 Results

### 2.1 Genome-wide identification and physicochemical analysis of the *CCoAOMT* family in pepper

Members of the *CaCCoAOMT* gene family were identified in the genome of the pepper cultivar ‘Zunla 1’ using HMMER-based searches. A total of 11 *CCoAOMT* genes were identified in the pepper genome and were systematically designated as *CaCCoAOMT1* through *CaCCoAOMT11*, according to their chromosomal positions (**Table 1**). The lengths of *CaCCoAOMT* proteins in pepper range from 143 to 380 amino acids. Their isoelectric points (pI) span from 4.90 to 9.47, and their molecular weights range from 16,461.92 Da to 43,000.98 Da. The instability index of these proteins varies between 27.31 and 45.22, while their aliphatic indices are between 94.34 and 113.68. The grand average of hydropathicity (GRAVY) values range from -0.247 to 0.070. Subcellular localization predictions indicate that *CaCCoAOMT1* is localized in the nucleus, whereas the other 10 *CaCCoAOMT* proteins are predominantly localized to both the cytoplasm and nucleus.

**Table 1:** Characterization of *CaCCoAOMT* family members in pepper.

Gene name	Gene ID	Number of Amino Acids	Theoretical p I	Molecular weight (Da)	Instability index	Aliphatic index	Average of hydropathicity (GRAVY)	Subcellular location
*CaCCoAOMT1*	Capana01g003929	380	9.47	43000.98	39.28	94.34	-0.119	nuclear
*CaCCoAOMT2*	Capana02g003073	242	5.28	27230.21	39.62	97.56	-0.236	Cytoplasmic and nuclear
*CaCCoAOMT3*	Capana02g003074	242	5.30	27222.23	37.64	97.56	-0.238	Cytoplasmic and nuclear
*CaCCoAOMT4*	Capana02g003075	242	5.29	27248.23	36.31	97.15	-0.247	Cytoplasmic and nuclear
*CaCCoAOMT5*	Capana04g002388	185	6.09	20812.13	32.76	102.81	-0.064	Cytoplasmic and nuclear
*CaCCoAOMT6*	Capana04g002394	143	5.33	16461.92	27.31	94.76	-0.195	Cytoplasmic and nuclear
*CaCCoAOMT7*	Capana04g002396	159	4.90	18345.94	43.22	103.58	-0.188	Cytoplasmic and nuclear
*CaCCoAOMT8*	Capana04g002397	154	9.14	17632.59	45.22	104.42	-0.006	Cytoplasmic and nuclear
*CaCCoAOMT9*	Capana08g002351	247	5.30	27822.91	31.32	98.34	-0.247	Cytoplasmic and nuclear
*CaCCoAOMT10*	Capana00g003512	152	5.35	16902.77	33.62	113.68	0.070	Cytoplasmic and nuclear
*CaCCoAOMT11*	Capana00g004448	282	5.79	31752.84	42.64	98.19	-0.081	Cytoplasmic and nuclear

### 2.2 Evolutionary analysis of *CCoAOMTs*

**Figure 1:**
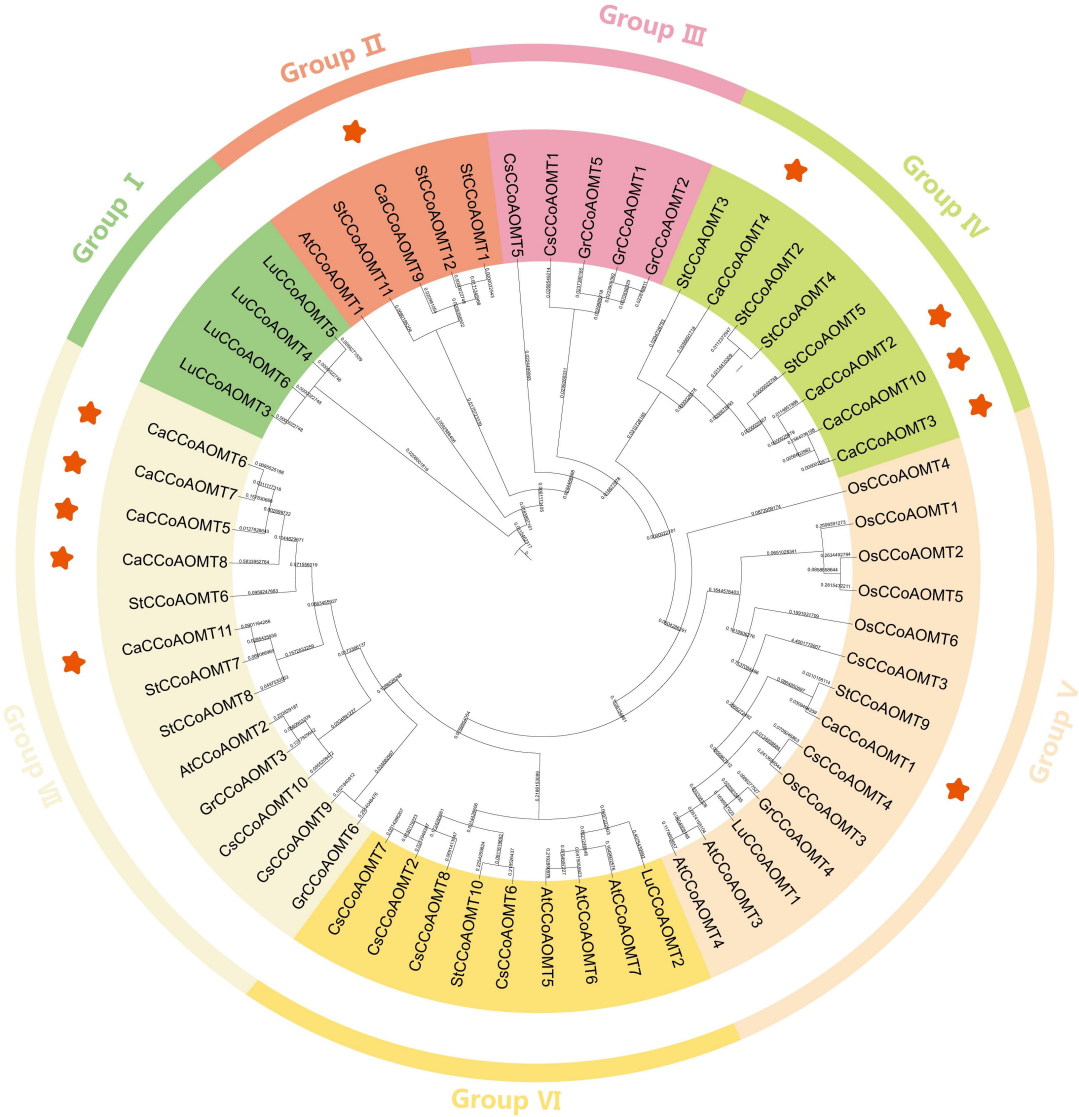
Phylogenetic analysis of *CCoAOMT* proteins from *Capsicum annuum* and other selected plant species. Species abbreviations: Ca (*Capsicum annuum*, highlighted in red), At (*Arabidopsis thaliana*), Os (*Oryza sativa*), Cs (*Camellia sinensis*), Gr (*Gossypium raimondii*), Lu (*Linum usitatissimum*), and St (*Solanum tuberosum*). The phylogenetic tree illustrates the evolutionary relationships and divergence of *CCoAOMT* proteins across these representative angiosperms.

To elucidate the evolutionary relationships within the *CCoAOMT* gene family, an un-rooted neighbor-joining phylogenetic tree was constructed based on 58 *CCoAOMT* protein sequences from seven angiosperm species: Capsicum annuum (11 members), Arabidopsis thaliana (7), Oryza sativa (6), Camellia sinensis (10), Gossypium raimondii (6), Linum usitatissimum (6), and Solanum tuberosum (12) (**Figure 1**; **Supplementary Table S1**). The phylogenetic analysis (**Figure 1**) delineated the *CCoAOMT* proteins into seven distinct clades, each characterized by distinct species distribution patterns. Notably, members of *CaCCoAOMT* were absent from clades I, III, and VI. The comparative analysis revealed that *CaCCoAOMT1* was grouped within clade V, alongside six rice homologs (*OsCCoAOMT1–6*), two Arabidopsis members (*AtCCoAOMT3* and *AtCCoAOMT4*), two tea plant homologs (*CsCCoAOMT3* and *CsCCoAOMT4*), and single representatives from flax (*LuCCoAOMT1*), cotton (*GrCCoAOMT4*), and potato (*StCCoAOMT9*). This grouping supports the hypothesis that *CaCCoAOMT1* shares a closer evolutionary relationship with rice and other species in clade V. Clade IV comprised four *CaCCoAOMT* members (*CaCCoAOMT2*, *CaCCoAOMT3*, *CaCCoAOMT4*, and *CaCCoAOMT10*) that clustered with three potato orthologs (*StCCoAOMT2*, *StCCoAOMT3*, and *StCCoAOMT4*). This observation provides further insight into the evolution of these genes in Capsicum and their relationship with other Solanaceae species. Additionally, *CaCCoAOMT9* formed a distinct subclade within clade II, in association with *AtCCoAOMT1* and three potato paralogs (*StCCoAOMT1*, *StCCoAOMT11*, and *StCCoAOMT12*). This clustering suggests specific evolutionary trajectories for *CaCCoAOMT9* within clade II. Notably, clade VII comprised five *CaCCoAOMT* proteins (*CaCCoAOMT5*, *CaCCoAOMT6*, *CaCCoAOMT7*, *CaCCoAOMT8*, and *CaCCoAOMT11*), which were clustered alongside three isoforms from potato (*StCCoAOMT6*, *StCCoAOMT7*, and *StCCoAOMT8*), a homolog from Arabidopsis (*AtCCoAOMT2*), two variants from the tea plant (*CsCCoAOMT9* and *CsCCoAOMT10*), and two homologs from cotton (*GrCCoAOMT3* and *GrCCoAOMT6*). These findings suggest a shared evolutionary origin for these *CaCCoAOMT* members, further supporting their potential functional similarities.

### 2.3 Chromosomal localization, intraspecific, and interspecific collinearity analysis of *CCoAOMT* genes

**Figure 2:**
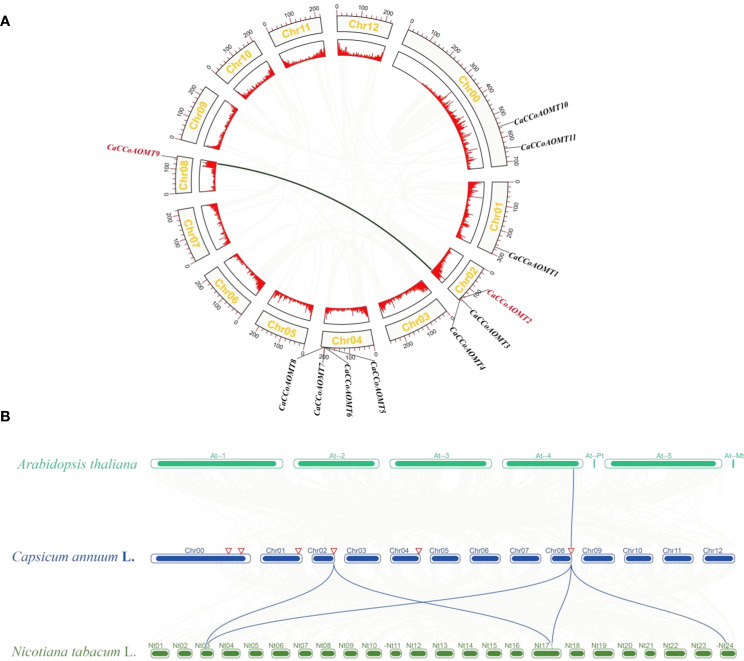
Collinearity analysis of *C annuum* and different species. **(A)** Intraspecific collinearity analysis of *CaCCoAOMT* genes. **(B)** Evolutionary relationship analysis between *C annuum* to *A thaliana*, and *N. tabacum*. Gray lines represent all synteny blocks identified between the genomes of different species.

Chromosomal localization analysis revealed that 9 *CaCCoAOMT* genes were physically mapped to four chromosomes (Chr01, Chr02, Chr04, and Chr08) in Capsicum annuum, displaying distinct distribution patterns (**Supplementary Figure S1**). Notably, two chromosomal regions exhibited prominent gene clustering: Chr04 harbored the highest proportion of genes (36.36%, 4 out of 11), followed by Chr02 (27.27%, 3 out of 11), while Chr01 and Chr08 each contained a single gene locus. Interestingly, *CaCCoAOMT10* and *CaCCoAOMT11* were not anchored to any chromosomal scaffold. Intra species collinearity found a collinear relationship between *CaCCoAOMT9* and *CaCCoAOMT2* (**Figure 2A**), indicating that their positions and arrangement in the genome may have high similarity or identical structural features. Through inter species collinearity analysis, a single orthologous gene pair was identified between Capsicum annuum and Arabidopsis thaliana, indicating that these two species have low conservation in genome structure and may have significant genome rearrangements or evolutionary differentiation (**Figure 2B**), this is consistent with the results of phylogenetic analysis (**Figure 1**). Whereas five conserved syntenic pairs were found between C. annuum and Nicotiana tabacum (**Figure 2B**). This suggests a greater divergence of C. annuum *CCoAOMTs* from Brassicaceae lineage genes, possibly due to lineage-specific expansions or differential gene loss.

### 2.4 Conserved motif composition and gene structure of *CaCCoAOMT* family members

**Figure 3:**
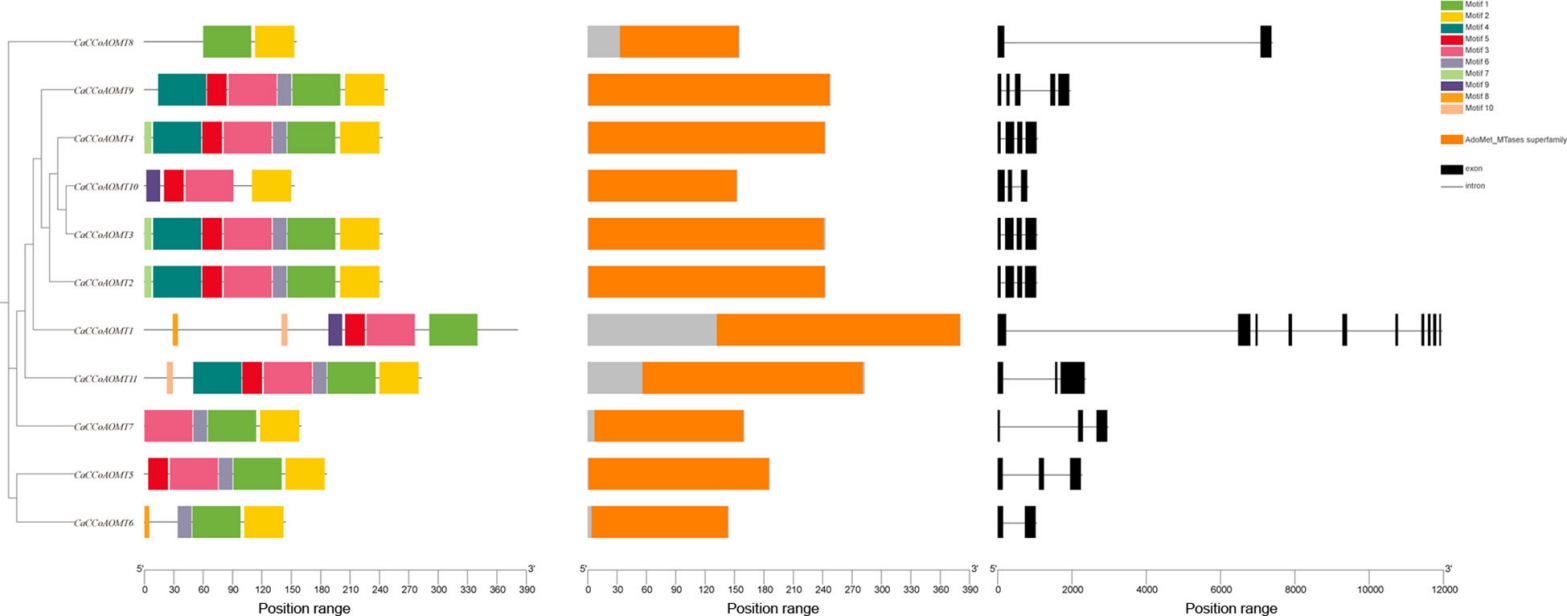
The phylogenetic relationship, conserved motifs, domain distribution, and exon–intron structures of the *CaCCoAOMTs*.

To further investigate the structural characteristics of *CaCCoAOMT* proteins, we performed a conserved motif analysis utilizing the MEME suite. Our analysis identified ten conserved motifs, labeled Motif 1 through Motif 10, across the *CaCCoAOMT* family (**Figure 3**), with each member exhibiting a single conserved AdoMet-MTase superfamily domain. Interestingly, the number of motifs per protein varied considerably, ranging from two to seven. Despite this variation, motif composition patterns were largely consistent within phylogenetic subgroups, corresponding to their clustering in the phylogenetic tree. Exon-intron structure analysis revealed substantial diversity in gene architecture. The number of introns within *CaCCoAOMT* genes varied from one (in *CaCCoAOMT6* and *CaCCoAOMT8*) to nine (in *CaCCoAOMT1*), with intermediate members containing two (*CaCCoAOMT5*, *CaCCoAOMT7*, *CaCCoAOMT10*, and *CaCCoAOMT11*), three (*CaCCoAOMT2*, *CaCCoAOMT3*, and *CaCCoAOMT4*), or four (*CaCCoAOMT9*) introns. The observed gradient in structural complexity, particularly the high intron content in *CaCCoAOMT1*, indicates that functional specialization and regulatory diversification have been influenced by evolutionary pressures within this gene family.

### 2.5 Cis-regulatory elements analysis of *CaCCoAOMT* genes

**Figure 4:**
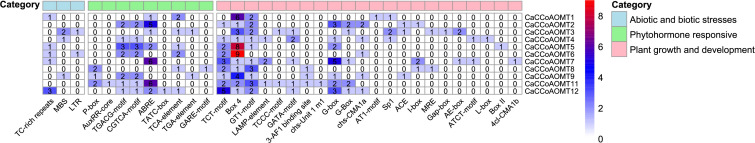
Analysis of cis-regulatory elements in the promoter region of *CaCCoAOMT* genes.

To elucidate the regulatory potential of the *CaCCoAOMT* genes, we conducted a systematic analysis of the cis-acting elements within their 2000 bp promoter regions. The ‘Plant growth and development’ category was predominantly composed of light-responsive elements, with Box4 being universally present across all *CaCCoAOMT* promoters. Notably, *CaCCoAOMT5* and *CaCCoAOMT6* exhibited the highest number of these elements. In addition to being crucial for regulating transcriptional activity under light, Box4 also play a critical role in responding to drought and salt stress, indicating that these genes may be involved in regulating plant light adaptation and stress resistance. In the ‘Phytohormone responsive’ category, ABA-responsive elements (ABRE) were identified in *CaCCoAOMT2*, *CaCCoAOMT7*, and *CaCCoAOMT11*. ABRE elements typically appear in the promoter regions of genes related to environmental stress response. These genes are activated by ABA signaling through the action of ABRE elements, helping plants cope with stress such as drought, salt, and low temperature. The ‘Abiotic and biotic stress’ category was the least represented, with MYB binding sites associated with drought inducibility (MBS) found in *CaCCoAOMT3*, *CaCCoAOMT4*, and *CaCCoAOMT9* (**Figure 4**). MYB transcription factors are closely related to the response of plants to environmental stresses such as drought, salinity, pests and diseases. MBS elements play an important role in the regulation of these genes. In addition, the role of MBS components in lignin synthesis, anthocyanin synthesis, and other pathways promotes the accumulation of these important chemicals in plants during growth and development.

### 2.6 Expression patterns of *CaCCoAOMT* genes across different tissues and fruit developmental stages

**Figure 5:**
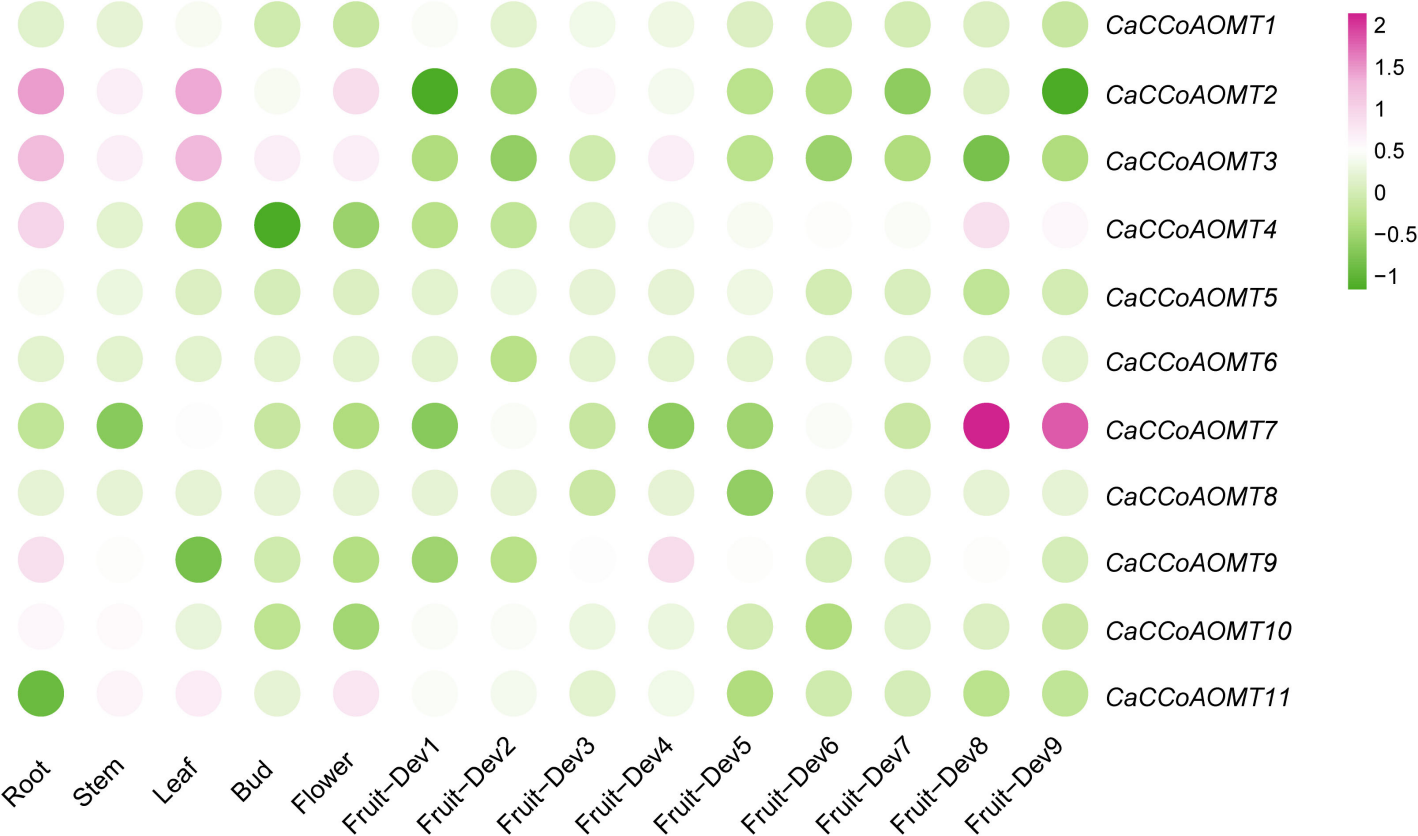
Heatmap representation of *CaCCoAOMT* gene expression across different tissues and developmental stages. Illumina RNA-seq data were used to assess *CaCCoAOMT* transcript levels in RNA samples from root, stem, leaf, bud, flower, and fruit tissues. The fruit developmental stages included nine phases: six pre-breaker stages (0–1 cm, 1–3 cm, 3–4 cm, 4–5 cm, and mature green fruit, ZL1-Dev1–5), the breaker stage (fruit turning red, ZL1-Dev6), and three post-breaker stages (3, 5, and 7 days after breaker, ZL1-Dev7–9). The FPKM values were log2-transformed, and the heatmap was generated using BAR Heat Mapper Plus software. The color bar at the bottom represents the log2-transformed values. Genes with high expression levels in the tissues are shown in red, while those with low expression are shown in green.

To investigate the potential functional roles of the *CaCCoAOMT* gene family in pepper, we systematically analyzed their expression profiles across different tissues and throughout various stages of fruit development. As illustrated in **Figure 5** and **Table S2**, *CaCCoAOMT1* demonstrated elevated expression levels in leaf tissues (ZL1-Leaf) and during the initial stage of fruit development (ZL1-F-Dev1). Regarding tissue-specific expression, *CaCCoAOMT2* and *CaCCoAOMT3* were predominantly expressed in roots (ZL1-Root) and leaves, whereas *CaCCoAOMT4* and *CaCCoAOMT5* exhibited strong root-specific expression. Importantly, *CaCCoAOMT6* showed no detectable expression in any of the tissues analyzed. In the context of fruit developmental stages, *CaCCoAOMT7* was markedly upregulated during the late fruit maturation phase (ZL1-F-Dev8), while *CaCCoAOMT8* was specifically activated at the mid-developmental stage (ZL1-F-Dev3). Furthermore, *CaCCoAOMT9* and *CaCCoAOMT10* displayed coordinated expression patterns in root tissues and at the fourth fruit developmental stage (ZL1-F-Dev4). *CaCCoAOMT11* was expressed in both leaf and floral tissues (ZL1-Flower). Collectively, these findings indicate that *CaCCoAOMT* genes are subject to dynamic and finely tuned regulation across various tissues and during fruit development, suggesting their diverse roles in organogenesis and developmental processes in pepper.

### 2.7 Morphology and structure of pepper under drought stress

**Figure 6:**
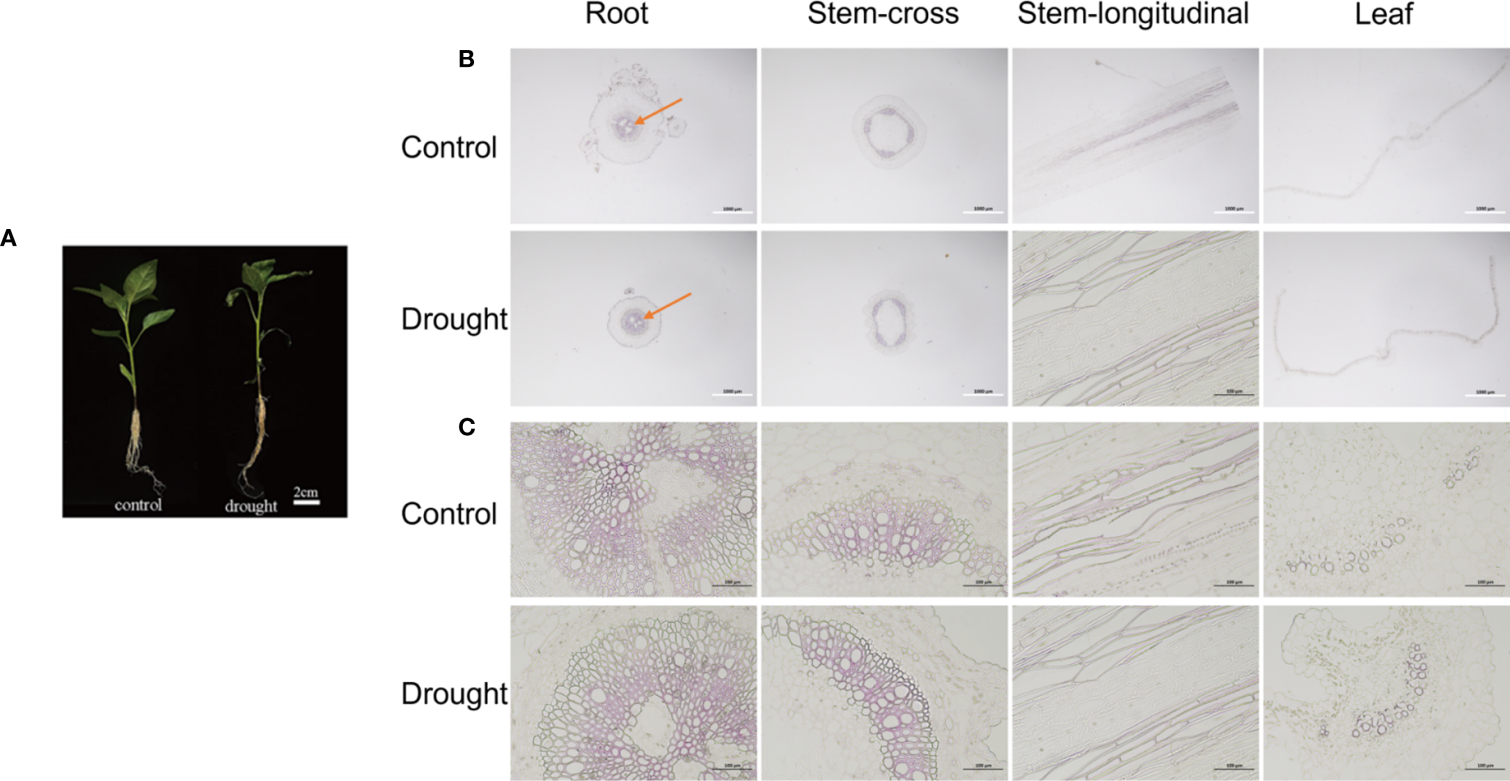
Morphology and structure of pepper root, stem and leaf under drought stress. **(A)** Morphology of pepper under control condition and drought stress. **(B, C)** Microstructure of root-cross section, stem-cross section, stem-longitudinal section and leaf-cross section under control condition and drought stress. The white scale is 1000 μm, and the black scale is 100 μm.

Drought stress elicited significant adaptive morphological and cytological alterations across various organs of pepper plants. Notably, the leaves exhibited pronounced curling, whereas the stems and roots demonstrated deformation (**Figure 6**). Microscopic analysis of root cross-sections revealed a substantial expansion of lignified regions, identified by red staining, in the drought-treated group compared to the control group, observable under both 20× and 200× magnification. In stem cross-sections, evidence of mechanical tissue damage was apparent, including shrinkage and rupture of epidermal cells, alongside intensified red coloration of xylem vessels in longitudinal sections. Leaf curling was associated with a disorganized arrangement of palisade tissue under 20× magnification, while 200× images revealed compromised cellular membrane integrity and marked plasmolysis.

### 2.8 Expression patterns of *CaCCoAOMTs* under drought stress

**Figure 7:**
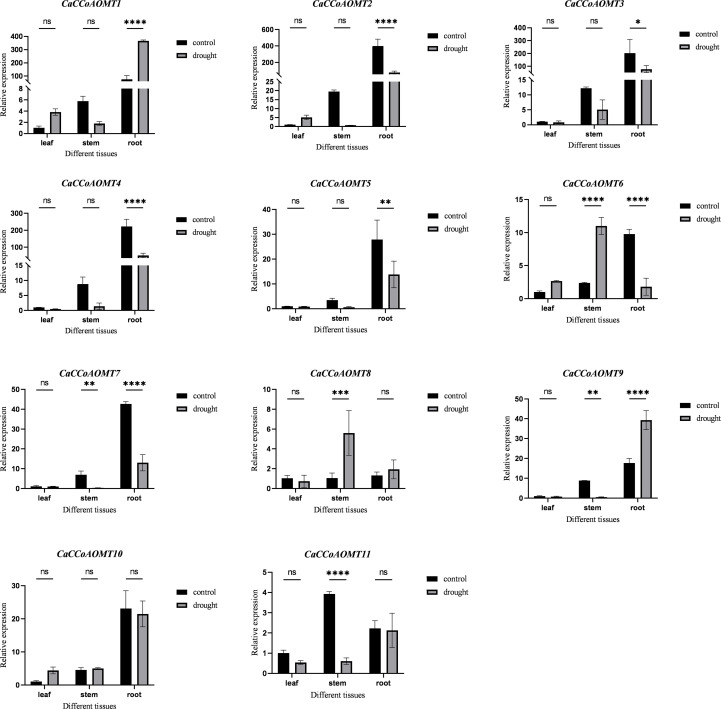
Expression analysis of *CaCCoAOMT* genes under drought stress using qRT-PCR. Plants were subjected to drought stress for two weeks. Data represent the mean ± SD of three biological replicates. "*" indicates p ≤ 0.05; "**" indicates p ≤ 0.005; "***" indicates p ≤ 0.0005; "****" indicates p ≤ 0.0001; "ns" indicates no significant difference.

We employed quantitative real-time PCR (qRT-PCR) to analyze the expression patterns of *CaCCoAOMT* genes under drought stress (**Figure 7**, **Table S3**). In the control group, most *CaCCoAOMT* genes exhibited higher basal expression levels in root tissues compared to stems and leaves. Under drought conditions, the expression of *CaCCoAOMT1* and *CaCCoAOMT9* in roots was significantly upregulated, while *CaCCoAOMT2*, *CaCCoAOMT3*, *CaCCoAOMT4*, *CaCCoAOMT5*, *CaCCoAOMT6*, and *CaCCoAOMT7* were markedly downregulated. In stems, drought treatment resulted in increased expression of *CaCCoAOMT6* and *CaCCoAOMT8*, whereas *CaCCoAOMT7*, *CaCCoAOMT9*, and *CaCCoAOMT11* consistently showed reduced expression. In leaf tissues, compared with the control, there was no significant change in the expression levels of 11 *CaCCoAOMT* induced by drought stress. The responses of *CaCCoAOMT* genes were consistent with phenotypic alterations under drought stress. The enhanced lignification observed in roots coincided with the significant upregulation of *CaCCoAOMT1* and *CaCCoAOMT9* in root tissues, suggesting that these genes actively contribute to drought-induced lignin biosynthesis and reinforcement of cell walls. Such reinforcement likely improves mechanical stability and reduces water loss by limiting apoplastic permeability. Conversely, the downregulation or unchanged expression of other *CaCCoAOMT* members under drought stress may reflect functionally divergent paralogs, some of which could act as regulatory isoforms with tissue-specific or developmental-stage–dependent roles, rather than as primary drought response factors. Pearson correlation analysis was performed on the RNAseq and qRT-PCR datasets to illustrate their relationship. The results indicate that there may be differences, which could be caused by various factors including batch effects, sample processing, or growth conditions (**Supplementary Table S4**). Overall, the strong and specific induction of *CaCCoAOMT1* and *CaCCoAOMT9* in roots after drought stress supports their identification as primary candidates for contributing to drought tolerance in pepper.

### 2.9 Subcellular localization of the *CaCCoAOMT* proteins

**Figure 8:**
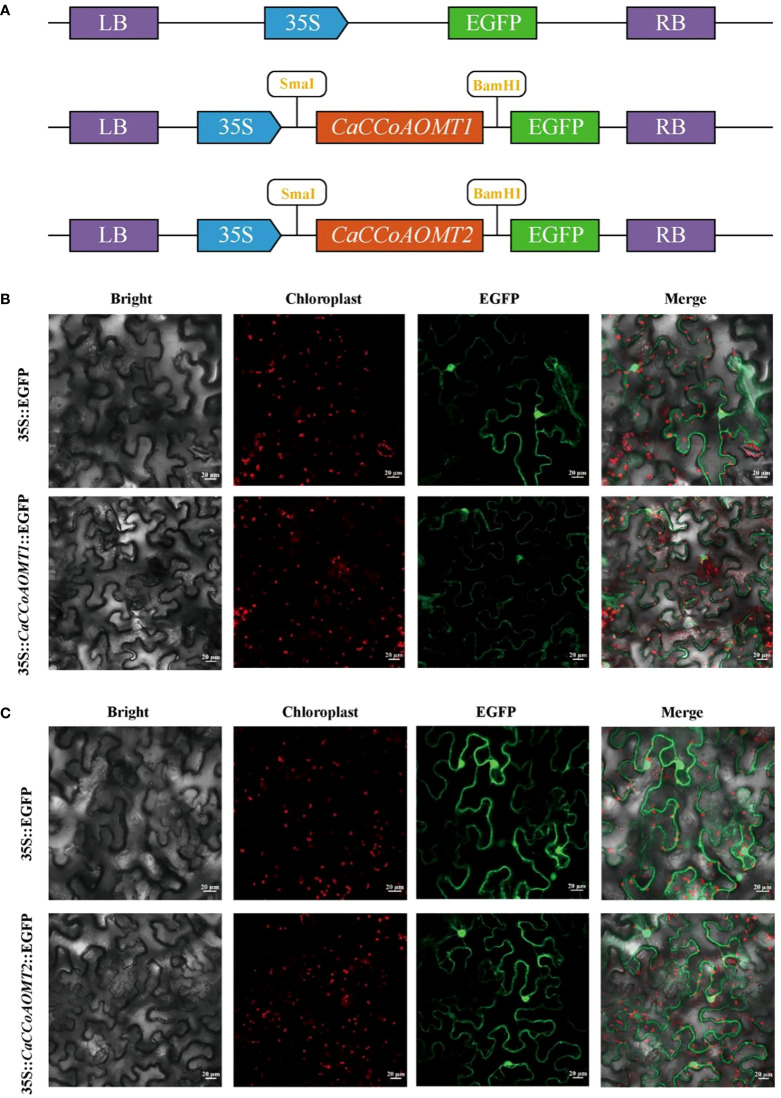
Subcellular localization of *CaCCoAOMT1* and *CaCCoAOMT2* by transient expression in the cells of tobacco leaves. **(A)** Schematic diagram of the pCAMBIA2300-35S-*CaCCoAOMT1*-EGFP and pCAMBIA2300-35S-*CaCCoAOMT2*-EGFP constructs. **(B)** Subcellular localization of *CaCCoAOMT1*-GFP fusion protein. **(C)** Subcellular localization of *CaCCoAOMT2*-GFP fusion protein.

To elucidate the subcellular localization of the *CaCCoAOMT1* and *CaCCoAOMT2* proteins in Capsicum annuum, we constructed the pCAMBIA2300-35S-*CaCCoAOMT1*-EGFP and pCAMBIA2300-35S-*CaCCoAOMT2*-EGFP plasmids (**Figure 8A**). These fusion proteins were transiently expressed in tobacco leaves via Agrobacterium-mediated transformation. The localization of the proteins was assessed using GFP fluorescence signals. The results indicated that both *CaCCoAOMT1* and *CaCCoAOMT2* were localized in the cytoplasm as well as the nucleus (**Figures 8B, C**). While the prediction suggested a different localization pattern (**Table 1**), the experimental data clearly showed that *CaCCoAOMT1* is distributed in both the cytoplasm and nucleus. Computational prediction tools often rely on amino acid sequence motifs and may not fully capture post-translational modifications, protein–protein interactions, or cell type–specific factors that affect protein localization in vivo. A GFP control, which lacked the protein fusion, was employed for comparative analysis.

### 2.10 Molecular docking of the *CaCCoAOMT* proteins

**Figure 9:**
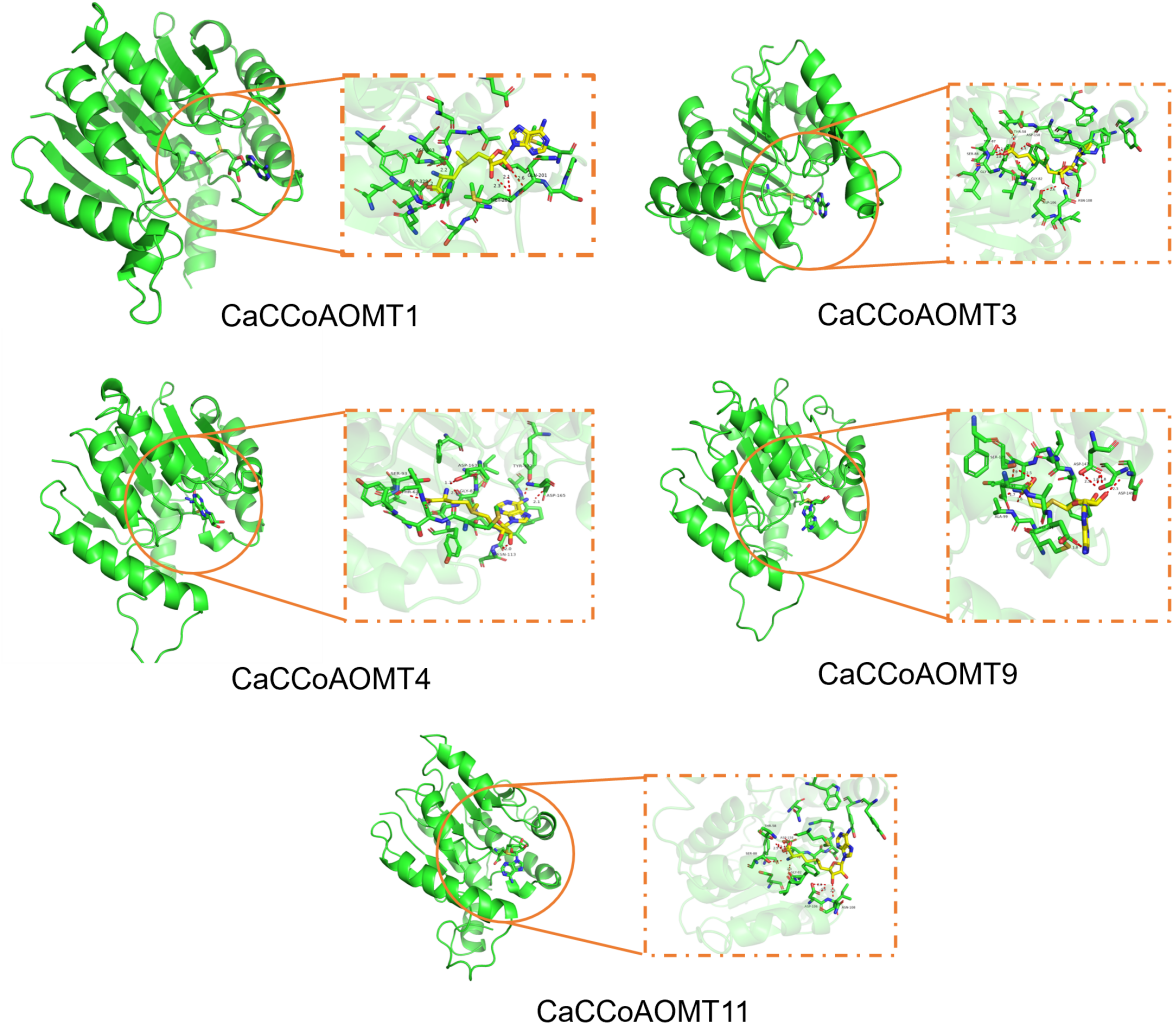
Docking simulations of *CCoAOMT1, CCoAOMT3, CCoAOMT4, CCoAOMT9, CCoAOMT11* proteins from chili peppers with the *S*-adenosylmethionine molecule.

Caffeoyl-CoA O-methyltransferase (*CCoAOMT*) is an important enzyme in plants, primarily involved in the methylation of caffeic acid, thereby generating a variety of crucial plant metabolites. *S*-adenosylmethionine (SAM), as a universal methyl donor, plays a key role in numerous methylation reactions. In this study, molecular docking analysis was performed to investigate the interactions between different *CCoAOMT* homologs and SAM, aiming to elucidate their potential roles in plant metabolism (**Figure 9**). The binding energies of *CCoAOMT1, CCoAOMT3, CCoAOMT4, CCoAOMT9*, and *CCoAOMT11* with SAM were calculated as -6.822 kcal/mol, -6.014 kcal/mol, -5.447 kcal/mol, -6.557 kcal/mol, and -7.435 kcal/mol, respectively. These results revealed the diversity of the *CCoAOMT* family in their interactions with SAM, with the differences in binding energies reflecting their functional divergence in catalytic activity and metabolic processes. Members with stronger binding affinities, such as *CCoAOMT1, CCoAOMT9*, and *CCoAOMT11*, are likely to play predominant roles in lignin biosynthesis and phenylpropanoid metabolism, whereas those with weaker affinities, such as *CCoAOMT4*, may serve more limited regulatory functions or exhibit tissue- or condition-specific activity. This is consistent with the observation that *CCoAOMT1* and *CCoAOMT9* are significantly upregulated under drought stress, indicating that they not only possess high catalytic potential at the enzyme–substrate interaction level but are also transcriptionally induced under stress conditions, thereby contributing to plant stress responses. As SAM is the methyl donor for a wide range of methyltransferase reactions, the efficiency of its interaction with *CCoAOMTs* directly influences the biosynthesis and accumulation of metabolic products. The differential affinities among *CCoAOMT* family members suggest that plants may achieve fine-tuned regulation of metabolic pathways through functional diversification of these enzymes, thereby enabling dynamic modulation of metabolite synthesis during different developmental stages or under varying environmental conditions.

## 3 Discussion

Lignin is a complex phenolic polymer predominantly located in the secondary cell walls of plants, where it plays an essential role in plant growth and development. It enhances the mechanical strength and water resistance of cell walls by cross-linking with cellulose and hemicellulose (Boudet et al., 1995). The biosynthesis of lignin involves numerous enzymatic reactions, which are regulated by a sophisticated gene regulatory network. Research indicates that lignin synthesis is vital not only for normal plant growth and development but also for the plant’s adaptive responses to environmental stresses (Choi et al., 2023). The *CCoAOMT* gene family is integral to the lignin biosynthesis pathway and is significantly involved in key physiological processes, including cell wall formation, disease defense, and responses to abiotic stress (Li et al., 2013; Sattler and FunnellHarris, 2013; Yang et al., 2021). This gene family has been extensively studied across various plant species, such as 12 in *Corchorus* (Kahie et al., 2023), 17 in *Camellia sinensis* (Wang et al., 2024), 9 in *Gossypium* (Ma et al., 2024), 12 in *Solanum tuberosum* (Peng et al., 2024),17 in *Dendrocalamus farinosus* (Wei et al., 2023), 5 in *Populus* (Zhao et al., 2022), as well as 12 in *Malus*, 8 in *Pyrus pyrifolia*, 15 in *Prunus persica* (Li et al., 2021), and 7 in *Sorghum bicolor* (Rakoczy et al., 2018). In this study, we identified 11 *CaCCoAOMT* genes based on the Zunla 1 genome. This number is comparable to that found in *Camellia sinensis* and *Solanum tuberosum*, but higher than in *Arabidopsis thaliana*, *Oryza sativa*, and *Gossypium raimondii* (**Table 1**, **Figure 1**).

The *CaCCoAOMT* genes demonstrate considerable variation in length, isoelectric point, molecular weight, and hydrophilicity, and such physicochemical diversity has been reported in other lignin-related O-methyltransferase families to underlie subfunctionalization and divergent expression profiles across tissues and developmental stages (Li et al., 2021; Zhao et al., 2022; Kahie et al., 2023; Wei et al., 2023; Ma et al., 2024; Peng et al., 2024; Wang et al., 2024). Combining the gene expression under drought stress (**Figure 7**), it suggests that structural divergence within the gene family may be linked to functional specialization, with certain isoforms (e.g., *CaCCoAOMT1* and *CaCCoAOMT9*) preferentially induced in roots during drought stress, while others exhibit downregulation or constitutive expression, potentially reflecting roles in developmental regulation rather than abiotic stress response. The molecular weight and isoelectric point of plant proteins are known to significantly influence their biochemical functions, a phenomenon extensively documented in plant proteomics research (Mohanta et al., 2019). For example, in *Arabidopsis*, the PERK gene family members exhibit substantial diversity in gene length, molecular weight, and isoelectric point, suggesting specialized functions among different members (Zhang et al., 2025). Moreover, the plant antioxidant system displays variations in subcellular localization and the functional capacity of enzymes encoded by different gene copies, further highlighting the structural and functional complexity of plant proteins (Bobrovskikh et al., 2020). At the gene structure level, most *CaCCoAOMT* genes possess the characteristic “AdoMet_MTases” domain, which is highly conserved in comparison to known *CCoAOMT* sequences from other species (Sattler and FunnellHarris, 2013; Li et al., 2021; Zhao et al., 2022; Kahie et al., 2023; Wei et al., 2023; Ma et al., 2024; Peng et al., 2024; Wang et al., 2024).

Chromosomal localization and homology analyses indicate that the expansion of the *CaCCoAOMT* gene family likely occurred through tandem duplication events. Specifically, the genes *CaCCoAOMT2*-*CaCCoAOMT4* and *CaCCoAOMT5*-*CaCCoAOMT8* form two clusters of tandemly repeated sequences. This mode of expansion is commonly observed in plant gene families. Tandem repeats, which refer to the sequential arrangement of genes in adjacent positions on chromosomes, play a significant role in plant evolution. For instance, in rice, the expansion of the cyclic nucleotide-gated channel (CNGC) gene family is associated with chromosomal segmentation and tandem repeats, with these genes playing crucial roles in responses to hormones, pathogens, and abiotic stresses (Nawaz et al., 2014). Similarly, in *Arabidopsis thaliana*, the expansion of the MORC gene family is linked to tandem repeats, contributing to epigenetic regulation and immune responses in plants (Dong et al., 2018). Inter-species collinearity analysis has revealed that *Capsicum annuum* shares numerous conserved homologous pairs with tobacco, but exhibits weaker collinearity with *Arabidopsis*. This suggests a higher degree of conservation of the *CCoAOMT* family within Solanaceae plants.

We conducted an analysis of the 2000 base pair promoter regions of *CaCCoAOMT* genes, identifying the cis-acting elements. Notably, light-responsive elements (Box4) were prevalent, particularly in *CaCCoAOMT5* and *CaCCoAOMT6*. Additionally, ABA-responsive elements (ABRE) were detected in *CaCCoAOMT2*, *CaCCoAOMT7*, and *CaCCoAOMT11*, while drought-related MYB binding sites (MBS) were observed in *CaCCoAOMT3*, *CaCCoAOMT4*, and *CaCCoAOMT9*, indicating their potential roles in stress response mechanisms. ABRE (ABA-responsive element) is a conserved cis-acting element in the promoter regions of plant genes related to ABA signal transduction, typically with the sequence PyACGTGGC. Under abiotic stresses such as drought, ABA levels increase, activating the ABRE-binding protein (AREB/ABF) family of transcription factors, which in turn promote the expression of ABA-responsive genes such as RD29B and Em. Upregulation of these genes helps plants regulate stomatal closure, synthesize osmotic adjustment substances, and produce antioxidant enzymes, thereby enhancing drought tolerance (Singh and Laxmi, 2015). The MYB transcription factor family is one of the largest in plants and plays a broad role in responses to abiotic stresses such as drought. MYB transcription factors regulate downstream gene expression by binding to MYB binding sites (MBS) in promoter regions, thereby modulating drought responses. For example, MYB44 has been shown to bind to MBS to repress the expression of the RD22 gene, thus regulating the ABA signaling pathway (Baldoni et al., 2015). These findings are consistent with previous studies on *Arabidopsis thaliana AtCCoAOMT1*, where expression is upregulated by ABA, MeJA, and salt stress, implicating its function in lignin biosynthesis and stress response pathways (Chun et al., 2019).

In this study, subcellular localization prediction suggested that *CaCCoAOMT1* is localized exclusively in the nucleus. However, transient expression analysis in tobacco leaf epidermal cells revealed that *CaCCoAOMT1* is localized in both the nucleus and cytoplasm (**Table 1**, **Figure 8**). Such discrepancies may arise because subcellular localization prediction algorithms often have inherent limitations, and their results do not always agree with experimental observations (Trofimov et al., 2019). In addition, as reported previously, subcellular localization results may vary when different receptor materials are used for transient expression assays, which can also lead to inconsistent findings (Lim et al., 2019; Zou et al., 2022).

Under drought conditions, plants generally increase lignin accumulation to enhance cell wall rigidity and minimize water loss (Bang et al., 2018; Yan et al., 2018). In this study, we found that drought stress significantly modified the anatomical structure of chili plants, particularly by increasing lignification in the roots and stems. Quantitative real-time PCR (qRT-PCR) analysis revealed that *CaCCoAOMT1* and *CaCCoAOMT9* were upregulated in the roots, with showing especially strong induction. These findings suggest a potential role for these genes in enhancing drought tolerance through the regulation of lignin biosynthesis. In *Arabidopsis*, the transgenic expression of *PaCCoAOMT1* and *PaCCoAOMT2* from ferns promoted lignin accumulation without affecting the levels of methylated flavonols, highlighting their crucial role in lignin production (Zhang et al., 2019). Similarly, in *Pinus radiata*, the suppression of *PrCCoAOMT* expression resulted in alterations in lignin content and composition, specifically in the proportions of H, C, and G units, which ultimately influenced plant mechanical strength and growth (Wagner et al., 2011). Collectively, these findings underscore the pivotal role of *CaCCoAOMT* genes in lignin biosynthesis and drought stress response, offering a valuable foundation for drought-resistant breeding and further functional characterization of these genes. In tomato, curated resources annotate Solyc02g093270 as a caffeoyl-CoA O-methyltransferase (*CCoAOMT*), and multiple studies report modulation of lignin-pathway genes under drought-related treatments in roots, aligning with our observation that drought enhances lignification (and *CCoAOMT* induction) in chili roots and stems (Xie et al., 2024). In potato, a recent genome-wide study identified 12 *StCCoAOMT* genes distributed on eight chromosomes, with collinearity and tandem/segmental duplications shaping the family; members were implicated in phenylpropanoid metabolism and stress-responsive regulation, consistent with lignification-linked roles under abiotic challenge (Peng et al., 2024). In addition, inter species collinearity analysis also found that *CCoAOMT* is more conserved among Solanaceae plants, and gene functions may also be more similar. Overall, Capsicum *CaCCoAOMT* genes likely share conserved, stress-responsive roles with *CCoAOMTs* of *Solanum lycopersicum* and *S. tuberosum*, reinforcing our inference that upregulated *CaCCoAOMTs* contribute to drought tolerance via lignin biosynthesis.

The results suggest that *CaCCoAOMT1* and *CaCCoAOMT9* are key candidates mediating lignin deposition in pepper roots under drought stress. Previous functional studies in *Arabidopsis thaliana*, *Pinus radiata*, and *Polypodiodes amoena* have demonstrated that altered expression of *CCoAOMT* genes directly modifies lignin content and composition, thereby influencing drought resistance and structural integrity. Thus, it is reasonable to hypothesize that the observed upregulation of *CaCCoAOMT1* and *CaCCoAOMT9* enhances drought tolerance in pepper through reinforcement of cell walls and reduced water loss. Future functional validation, such as CRISPR/Cas9-mediated knockout, virus-induced gene silencing, or overexpression in transgenic pepper lines, combined with lignin quantification and drought survival assays, will be essential to confirm their mechanistic contribution.

## 4 Materials and methods

### 4.1 Plant materials and growth conditions

Seedlings of Capsicum annuum cultivar ‘ZunLa 1’, preserved in the Germplasm Resource Bank of the Pepper Research Institute, Guizhou Academy of Agricultural Sciences, were used as experimental materials. The selected seeds were surface-sterilized and then cultivated in pots (20 cm diameter × 20 cm height) filled with nutrient-rich substrate. Plants were grown in a controlled greenhouse under identical environmental conditions for both treatments: a 30/26°C (day/night) temperature regime, 16/8-hour light/dark photoperiod, and ~60–65% relative humidity. After 20 days of growth, seedlings were divided into two groups, with 15 plants per treatment (three biological replicates, each replicate consisting of 5 plants). For the drought-stressed group, irrigation was withheld for 14 days to impose natural drought conditions. Drought severity was verified by measuring soil volumetric water content using a soil moisture probe, which dropped from ~75% to ~30% at the end of the treatment. For the well-watered control group, plants were maintained under the same environmental conditions but irrigated every 2 days to sustain the substrate at ~75–80% of field capacity. At the end of the 14-day treatment, roots, stems, and leaves were collected from both control and drought-stressed plants, immediately flash-frozen in liquid nitrogen, and stored at –80°C for subsequent RNA extraction and expression analysis.

### 4.2 Identification of *CaCCoAOMT* genes

The pepper genome data was downloaded from the Sol Genomics Network (https://www.sgn.cornell.edu/organism/Capsicum_annuum/genome) (Qin et al., 2014), and the HMM model file (PF01596) for the *CaCCoAOMT* gene family in pepper was obtained from the Pfam (v 37.1) website (https://pfam.xfam.org/) (El-Gebali et al., 2019). The HMMER (v 3.3) software was used to search for *CCoAOMT* proteins within the pepper protein sequences (Potter et al., 2018). Domain annotation of the sequences was performed using the pfamscan tool and the SMART database, with the sequences containing the “AdoMet_MTases” domain being identified as the final candidate sequences for the *CaCCoAOMT* gene family.

### 4.3 Bioinformatic analysis of the *CaCCoAOMT* gene family

The online tool ExPASy (https://web.expasy.org/) was used to analyze the physicochemical properties of the proteins, while ProtComp v. 9.0 (http://linux1.softberry.com/berry.phtml) was employed to predict their subcellular localization. Multiple sequence alignment analyses of the *CCoAOMT* protein sequences from *C. annuum* and other species were performed (Wu et al., 2024). A phylogenetic tree was constructed for the *CCoAOMT* genes of *D. farinosus* using the Clustal W program in MEGA 7.0 software with the neighbor-joining (NJ) method and 1000 bootstrap replications (Li et al., 2025). The *CaCCoAOMT* protein sequences were uploaded to the NCBI Conserved Domain Database and the MEME Suite to obtain information on conserved domains and motif patterns (Bailey et al., 2015). The promoter regions, consisting of 2000 bp upstream of the start codon of the *CaCCoAOMT* genes, were extracted using the TBtools (v2.142) GXF Sequences Extract tool (Chen et al., 2023). The extracted sequences were then submitted to the PlantCARE database for analysis of cis-acting regulatory elements (Lescot et al., 2002). Based on the 11 identified *CaCCoAOMT* sequences and the genome annotation file of pepper (Zunla-1), collinearity analysis was performed using the TBtools (v2.142) One Step McscanX tool with the specified parameters. The homologous relationships between *CaCCoAOMT* genes and those in *Arabidopsis thaliana* and *Solanum lycopersicum* were analyzed using MCScanX (Wang et al., 2012). Segmental and tandem duplication events in the *CaCCoAOMT* genes were identified and visualized using TBtools-II v2.142. Subcellular localization prediction of *CaCCoAOMT* was conducted using the online website wolf-psort (https://www.genscript.com/wolf-psort.html).

### 4.4 Tissue expression pattern analysis of the *CaCCoAOMT* gene family

The transcriptome annotation files of *C. annuum* (Zunla-1) at different developmental stages were retrieved from the GEO database (https://www.ncbi.nlm.nih.gov/geo/, accession number GSE45037), including root, stem, leaf, bud, flower, and different fruit development stages. The TBtools-II v2.142 software was used to generate expression heatmaps for the *CaCCoAOMT* gene family in pepper.

### 4.5 Morphology and structure of pepper under drought stress

Roots, stems, and leaves from representative drought-treated and control pepper plants were immediately placed in FAA fixative (70% ethanol: formaldehyde: glacial acetic acid = 90:5:5) and fixed at 4*°C* for 24–48 h. After fixation, samples were dehydrated through a graded ethanol series and embedded in Technovit 7100 resin to prepare hard tissue sections. Embedded blocks were sectioned into 5–8 µm slices using a Leica RM2265 microtome. After de-plastification, the sections were stained with phloroglucinol to detect the distribution and degree of lignification. Sections were de-plastified and stained with 1% phloroglucinol in ethanol for 2–5 min, followed by a few drops of concentrated HCl to induce coloration. Lignified cell walls appeared red under light microscopy. Slides were temporarily mounted with neutral balsam and observed under an Olympus BX53 microscope at 20× and 200× magnification (Gritsch et al., 2015). For each treatment, three biological replicates (three independent plants per group) were processed. From each replicate, at least five sections per tissue type (root, stem, leaf) were examined, and representative images were captured.

### 4.6 RNA extraction and quantitative RT-PCR analysis

Total RNA was extracted from the roots, stems, and leaves of pepper plants under control and drought stress conditions using the TRIzol™ Reagent Kit (Invitrogen). cDNA was synthesized from 1 μg of RNA using the HiPro™ (H-) 1st Strand cDNA Synthesis Kit with gDNA Eraser (Beijing PruTone). Primers for the *CaCCoAOMT* genes were designed based on their CDS sequences (**Supplementary Table S2**) and synthesized by Genewiz Biotechnology (Shanghai, China), with *CaEIF5A2* used as the internal reference gene. qRT-PCR was conducted using the ProQ™ qPCR EvaGreen Master Mix (PruTone), with an ABI ViiA7 Real-Time PCR System (Life Technologies, USA) as the PCR instrument. The reaction volume was 25 μL, consisting of 2 μL cDNA, 0.5 μL of each forward and reverse primer, 10 μL of ProQ™ qPCR EvaGreen Master Mix, and ddH2O to a final volume. The PCR conditions were as follows: initial denaturation at 94°C for 3 minutes, followed by 40 cycles of 94°C for 10 seconds, 60°C for 20 seconds, and 72°C for 30 seconds. The relative expression levels of genes were calculated using the 2^-ΔΔCT^ method (Livak and Schmittgen, 2001). Gene expression levels were analyzed using Excel 2019, statistical significance was assessed using SPSS 19.0, differences between control and drought-stressed groups were tested using independent-samples t-test (*p* < 0.05), and graphs were generated using GraphPad Prism 8.0.

### 4.7 Subcellular localization of *CCoAOMT* proteins

To determine the subcellular localization of *CaCCoAOMT1* and *CaCCoAOMT2*, we constructed the pCAMBIA2300-35S-*CaCCoAOMT1*-EGFP and pCAMBIA2300-35S-*CaCCoAOMT2*-EGFP plasmids. These fusion proteins were transiently expressed in tobacco leaves via Agrobacterium-mediated transformation. GFP fluorescence signals were used to observe the subcellular localization of the proteins. The localization was examined using a confocal microscope, with GFP signals visualized at an excitation wavelength of 488 nm.

### 4.8 Molecular docking

The PDB structure of the *CCoAOMT* protein was predicted using Phyre2 (https://www.sbg.bio.ic.ac.uk/~phyre2/html/page.cgi?id=index). Molecular docking was performed with AutoDock software. The docking results were visualized using PyMOL.

## 5 Conclusions

In this study, we systematically identified and analyzed 11 *CCoAOMT* genes in the *Capsicum annuum* genome, designated *CaCCoAOMT1* to *CaCCoAOMT11*. Analysis of their physicochemical properties, gene structures, conserved motifs, subcellular localization, and evolutionary relationships revealed the diversity and expansion of the *CaCCoAOMT* gene family in pepper. Transcriptome data analysis showed that these genes exhibit distinct expression patterns across different tissues and developmental stages. Furthermore, their responses to drought stress were validated by qRT-PCR. *CaCCoAOMT1* and *CaCCoAOMT11* are the most important candidate genes for pepper drought resistance. In the future, the precise functions of *CaCCoAOMT1* and *CaCCoAOMT9* in drought resistance response can be further revealed, especially in lignin synthesis, plant cell wall reinforcement, and plant water regulation. By utilizing the drought resistance characteristics of these genes, new varieties with stronger drought resistance can be developed. Subcellular localization experiments demonstrated that *CaCCoAOMT1* and *CaCCoAOMT2* proteins are mainly localized in both the cytoplasm and nucleus. While our results identify *CaCCoAOMT1* and *CaCCoAOMT9* as prime candidates for drought resistance, future studies employing functional assays (e.g., gene silencing, overexpression, lignin quantification, and drought survival tests) will be crucial to validate their mechanistic roles. This study thus lays an important foundation for subsequent functional research and offers valuable gene resources for improving drought tolerance in pepper through molecular breeding.

## Data Availability

The original contributions presented in the study are included in the article/**Supplementary Material**. Further inquiries can be directed to the corresponding authors.
