# Supplementary figures and images for: Genome-wide identification and expression analysis of CCoAOMT genes in Capsicum annuum L. under drought stress
Source: Front Plant Sci. 2025 Sep 19;16:1654390. doi: 10.3389/fpls.2025.1654390 (PMC12494069; doi:10.3389/fpls.2025.1654390)

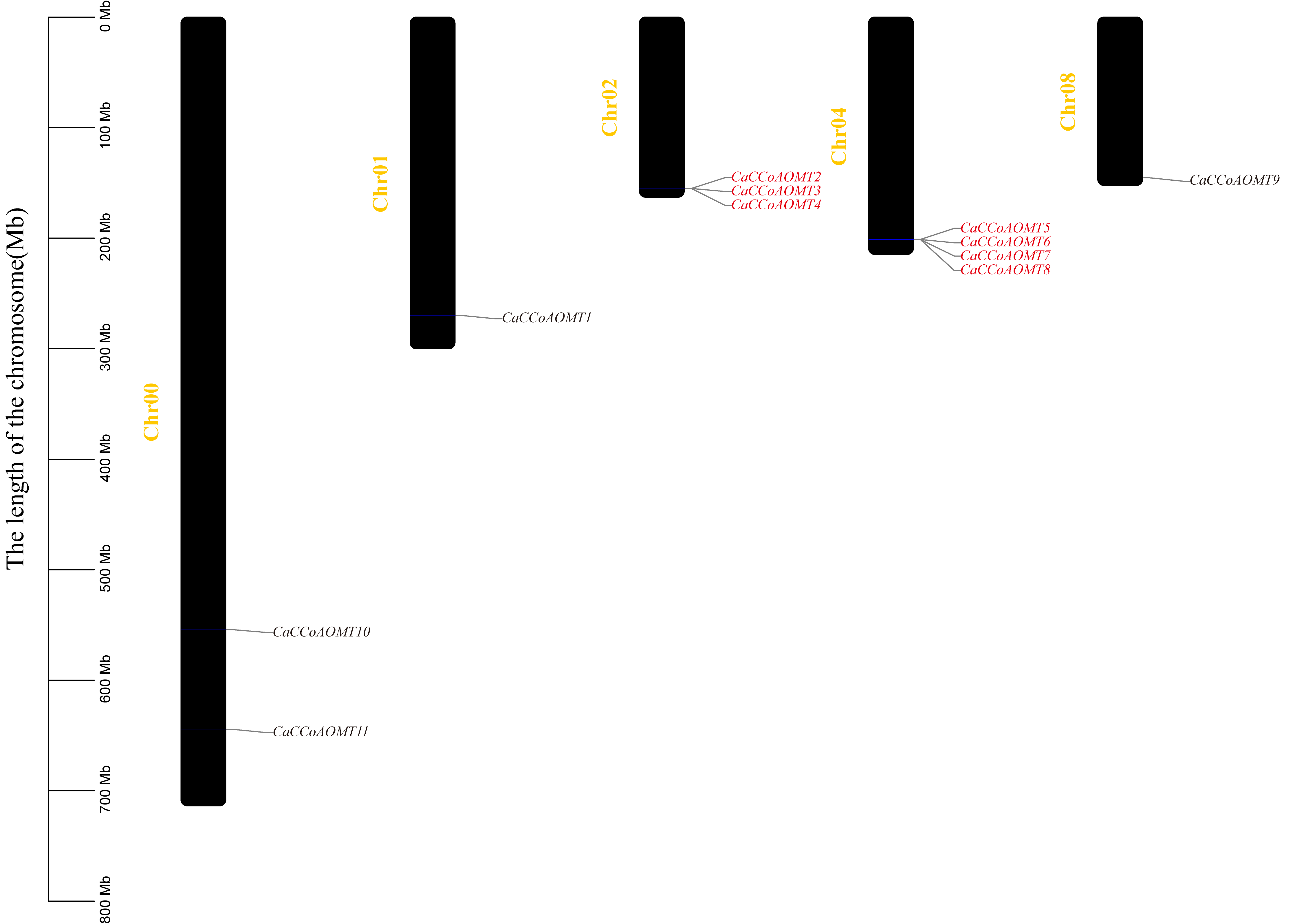

Supplement: Supplementary file 1 [file DataSheet1.zip › Supplementary figure S1-Chromosome Localization of CaCCoAOMT.tif]
